# Higher Genetically Predicted Triglycerides, LDL, and HDL Increase the Vitamin D Deficiency: A Mendelian Randomization Study

**DOI:** 10.3389/fnut.2022.862942

**Published:** 2022-05-03

**Authors:** Zhe Lu, Yang Jiao, Jun Li

**Affiliations:** ^1^Ultrasonic Diagnosis Department, The 946th Hospital of P.L.A, Yili Group, Hohhot, China; ^2^Ultrasonic Diagnosis Department, Production and Construction Corps Hospital, Urumqi, China; ^3^Department of Pathology, School of Basic Medical Sciences, Southern Medical University, Guangzhou, China

**Keywords:** HDL, vitamin D deficiency, mendelian randomization, triacylglycerol, LDL

## Abstract

**Introduction:**

It has been proven that high body mass index (BMI) levels can cause vitamin D deficiency, but the mechanism is still unclear. Therefore, this study attempts to explain this phenomenon from the perspective of blood lipid by using mendelian randomization (MR).

**Methods:**

Genome-wide association studies (GWAS) summary datasets for serum lipids were obtained from the Global Lipids Genetics Consortium (GLGC). Vitamin D deficiency outcome data were acquired from the UK Biobank samples. Single-variable MR (SVMR) and multi-variable MR (MVMR) analyses were conducted using the TwoSampleMR package based on R 4.0.3. The four main methods were the random-effect inverse-variance weighted (IVW), MR-Egger, weighted-median method, and weighted mode.

**Results:**

In the SVMR of serum lipid/apolipoprotein levels on serum vitamin D level, it was found that elevated serum triacylglycerol (IVW, OR = 0.85, 95%CI:0.81–0.89, *P* < 0.001), low-density lipoprotein (LDL) (IVW, OR = 0.93, 95%CI:0.90–0.95, *P* < 0.001), and high-density lipoprotein (HDL) (IVW, OR = 0.95, 95%CI:0.91–0.98, *P* < 0.001) levels all had a causal relationship with vitamin D deficiency, but significant pleiotropy was detected in the triacylglycerol (*P* = 0.001) and HDL (*P* = 0.003) analysis. MVMR analysis results were consistent with SVMR.

**Conclusion:**

By using single-variable mendelian randomization and multi-variable mendelian randomization methods, we identified that the elevated serum triacylglycerol, LDL, and HDL levels all had a causal relationship with vitamin D deficiency. Taking into account the significant pleiotropy demonstrated in this study, the conclusions of this study should be treated with caution.

## Introduction

The primary role of vitamin D is to participate in calcium and phosphorus metabolism, promote calcium and phosphorus absorption in the intestine, and bone calcification (1). Active vitamin D such as [1,25(OH)2D3], which is the primary circulating form of vitamin D and is the best way to monitor vitamin D levels, can act on the nucleus of small intestinal mucosal cells to promote the biosynthesis of calcium transport-related proteins, thereby accelerating the absorption of calcium (2). Vitamin D promotes phosphorus absorption, possibly, by indirectly promoting the absorption of calcium. Therefore, the effect of active vitamin D on calcium and phosphorus metabolism increases blood calcium and blood phosphorus, so that the levels of plasma calcium and plasma phosphorus reach homeostasis (3).

Vitamin D deficiency usually leads to diseases related to calcium and phosphorus metabolism, such as osteoporosis, rickets, and kidney stones (4). Inappropriate vitamin D supplementation may lead to other diseases such as kidney stones (5). Therefore, it is a practical study to clarify the risk factors that lead to vitamin D deficiency. Considering the current increase in the incidence of overweight in developed countries, obesity seems to be a factor worthy of attention among the many risk factors related to vitamin D deficiency that has been clarified (6–9). A systematic review after pooling 23 primary studies found that the prevalence of vitamin D deficiency was 35% higher in obese subjects compared to the eutrophic group. However, the underlying mechanism has not yet been clarified (10).

It is worth noting that some studies have found that there were significant correlations between vitamin D supplementation and serum lipid changes (11). Some articles have discussed the association between lipid profiles and Vitamin D levels and found some significant relationships with lipid profiles (cholesterol, LDL, and HDL) during the different seasons. Still, there was a previously published Mendelian Randomization Study, which pointed out that a higher BMI can lead to a decrease in vitamin D. Therefore, we were more inclined to believe that elevated lipid levels in the serum can lead to a decrease in vitamin D levels (12, 13). Based on the conclusions of the existing literature reports, we put forward the hypothesis that when the lipid metabolism of the population is disordered, the disorder of vitamin D metabolism is more likely to occur. There is still no published literature on this hypothesis. Therefore, in this analysis, we tried to use Mendelian randomization as a research method to clarify the causal relationship between the increase in serum lipids and the decrease in vitamin D (14).

A randomized controlled trial is the best trial protocol to discuss the causal relationship between a risk factor and an outcome event. However, RCT research is almost impossible in etiology research due to ethical and many other limitations. Mendelian randomization, which uses genetic information as instrumental variables, has been widely used in etiological studies (15). MR utilizes variant stochastic genetic mechanisms to establish random assignment before outcome events and, thus, is relatively independent of confounders as a tool to assess the causal impact of risk factor exposure on health outcomes of interest (16). Multivariable Mendelian randomization (MVMR) is a recently developed approach that allows simultaneous assessment of individual, but related exposures by incorporating genetic variations from each risk factor into the same model (17). Considering the completeness and accessibility of the data, the serum lipid level only examined three serum lipids: triglycerides (TG), low-density lipoprotein (LDL), and high-density lipoprotein (HDL). Only [1,25(OH)2D3] was discussed in this analysis.

## METHODS

### Data Sources

We selected the largest lipid-related GWAS database independent of Ukbiobank from The Global Lipids Genetics Consortium (GLGC) datasets (Largest database outside of Ukbiobank, ID: ieu-a-300 for LDL based on 173,082 patients, ieu-a-299 for HDL based on 187,167 patients, ieu-a-302 for TG based on 177,861 patients) for exposure (16, 18, 19). The GWAS summary statistic data for serum [1,25(OH)2D3] level from a previously published study based on UK Biobank samples was used as outcome datasets (https://gwas.mrcieu.ac.uk/datasets/ebi-a-GCST90000615/) (20). F-statistics of all the included GWAS were larger than 10. In this way, the basic assumption that the original GWAS study included in MR analysis was satisfied.

### Statistical Analyses

Two-sample MR and MVMR analyses were conducted with the TwoSampleMR package based on R 4.0.3 (https://github.com/MRCIEU/TwoSampleMR) (21). Reported significant associated single nucleotide polymorphisms (SNPs) for outcome and exposures were included in the MR analysis as mediators. However, SNPs in linkage disequilibrium (LD) (*r*^2^ = 0.01, 10,000 kb) using the 1,000 genomes reference panel and palindromic SNPs should be excluded (16).

The MR analyses were mainly conducted using a 2-sample inverse variance weighted (IVW) method. This method consisted of meta-analyzing SNP-specific Wald ratios between the effect outcome [β_25(OH)D_] and exposure (β _lipids_) using a random-effects inverse variance method that weights each ratio by its standard error while accounting for possible heterogeneity in measures. Furthermore, MR-Egger provides an unbiased effect even though the instrumental genetic variables (GIVs) influence the outcome not only through the exposures (22). The Median-based estimator only needs half of the SNPs to be validated (23). Heterogeneity amongst GIVs is an indicator of potential violations of MR core assumptions. It was calculated for IVW and MR-Egger and used to navigate with horizontal pleiotropy models (24). Besides, we used the intercept from MR-Egger to test the directional pleiotropy. MR-Egger could also be used as an adjustment method for significant pleiotropy (25). To avoid potential bias caused by multiple exposures on the outcome, multivariable MR analysis (MVMR) based on IVW mothed was applied in this analysis to identify independent SNPs and calculate an adjusted estimate (26).

All statistical analyses mentioned above were achieved through R v.4.0.3 (www.r-project.org), *TwoSampleMR, ieugwasr*, and *data. The table* was the main R packages used in this study. All the reported *P*-values were 2-sided, and significance was indicated as *P* < 0.05. Sample codes of this analysis can be achieved at https://mrcieu.github.io/TwoSampleMR/index.html (27).

## Results

### Single Variable MR Analysis of Serum Lipid/Apolipoprotein Levels on Serum Vitamin D Level

**Figure 1:**
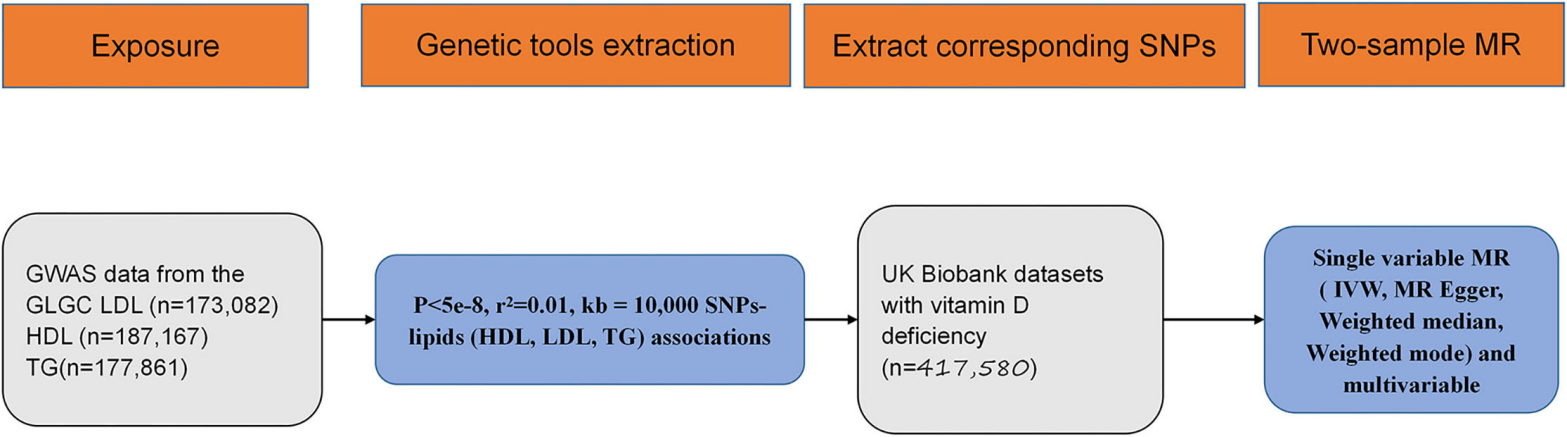
Flow diagram for eligible single nucleotide polymorphism (SNPs) selection for exposure and outcome. SNP, single nucleotide polymorphism; GWAS, genome wide association study; MR, mendelian randomization; HDL, high density lipoprotein; LDL, low density lipoprotein; TG, triglycerides; IVW, inverse variance weighted.

Exposure and outcome associated with significant SNPs' screening flowchart were displayed in Figure 1. According to the published study, in which we obtained outcome GWAS summary data, vitamin D deficiency was defined as 25-hydroxyvitamin D < 25 nmol/L (20). Based on GLGC datasets, out of 2,437,752 SNPs, 74, 85, and 54 significantly associated SNPs were identified between LDL, HDL, TG, and vitamin D deficiency. The unit of summary data was SD, and the SD of LDL was 38.67 mg/dL (https://gwas.mrcieu.ac.uk/datasets/ieu-a-300/), the SD of HDL was 15.51 mg/dL (https://gwas.mrcieu.ac.uk/datasets/ieu-a-299/), and the SD of TG was 90.72 mg/dL (https://gwas.mrcieu.ac.uk/datasets/ieu-a-302/) (28). In this analysis, all the OR means odds ratio caused by per 1-standard-deviation–higher trait.

**Figure 2:**
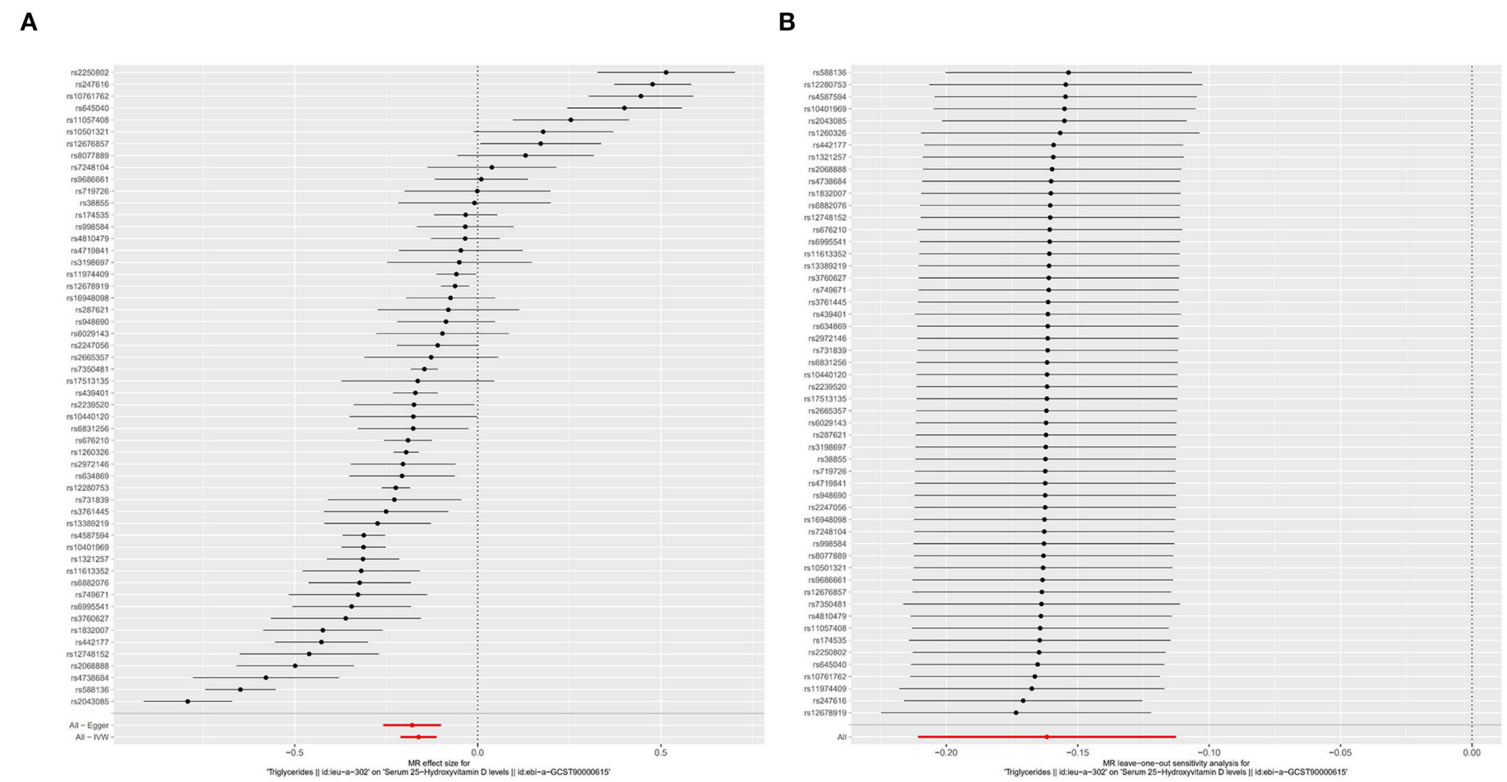
Forest plot of included SNPs in the mendelian randomization analysis for casual effect of triglycerides on vitamin D deficiency. **(A)** Meta-analysis of SNPs' effects on vitamin D deficiency based on inverse variance weighted (IVW) method with random effect model. **(B)** Sensitivity analysis by omitting every SNP.

**Figure 3:**
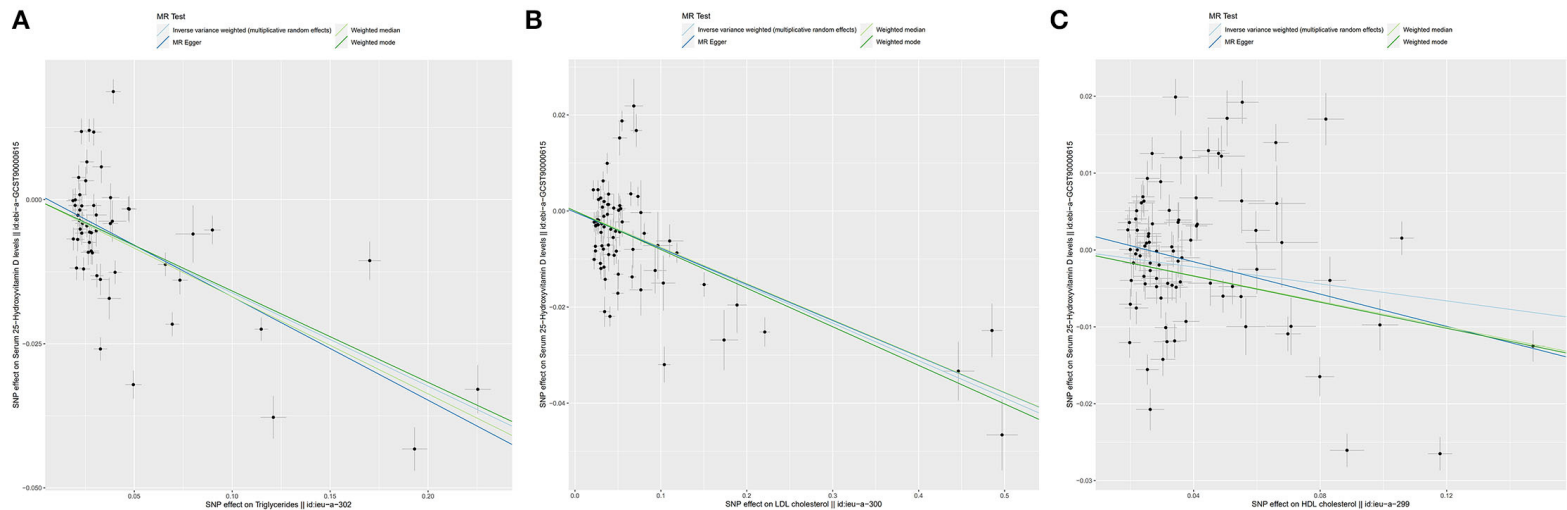
The scatter plots for Mendelian Randomization (MR) analyses of the causal effect of lipid profile on vitamin D deficiency. Analyses were conducted by using the conventional IVW, MR-Egger, weighted median MR, weighted mode MR. The slope of each line corresponding to the estimated MR effect per method. **(A)** Triglycerides. **(B)** LDL. **(C)** HDL. LDL, Low-density lipoprotein; HDL, High density lipoprotein.

In the SVMR analysis, it was found that elevated serum TG concentration could cause serum [1,25(OH)2D3] level to decrease (Figure 2A, OR based on IVW:0.85, 95%CI:0.81-0.89, *P* <0.001), further sensitivity analysis found that the synthesized result was stable (Figure 2B), Q test indicated that the heterogeneity was significant (**Table 2**, Q_IVW_=835.3, *P* < 0.001), considering that a total of 54 SNPs were included for data merging, thus, this is reasonable. MR-egger Intercept test found significant pleiotropy in the SVMR between serum TG and serum vitamin D (*P*__*i*_ntercept_ = 0.001). MR-egger method was applied and found that after the pluripotency adjustment, the causal relationship between elevated TG level and decreased [1,25(OH)2D3] level was still significant (Table 1), MR-egger part, OR: 0.84, 95%CI: 0.77–0.90, *P* < 0.001). The further weighted median MR method (OR: 0.84, 95%CI: 0.82–0.87, *P* < 0.001) and the weighted mode MR method (OR:0.85 95%CI:0.83–0.88, *P* < 0.001) were also conducted, and all the results were stable (Table 1, Figure 3A).

**Table 1:** Associations based on univariate MR between genetically predicted circulating lipid levels and serum vitamin D level are estimates using summary data from UKB and GLGC datasets.

**Lipids**	**IVW**		**MR-Egger**			**Weighted median**	**Weighted mode**
	**OR (95% CI)** **(***P***-value)**	**Q** **(***P***-value)**	**OR (95% CI)** **(***P***-value)**	**Q** **(***P***-value)**	**Intercept** **(***P***-value)**	**OR (95% CI)** **(***P***-value)**	**OR (95% CI)** **(***P***-value)**
TG (SNP = 54)	0.85 (0.81, 0.89) (<0.001)	835.3 (<0.001)	0.84 (0.77, 0.90) (<0.001)	830.2 (<0.001)	0.001	0.84 (0.82, 0.87) (<0.001)	0.85 (0.83, 0.88) (<0.001)
LDL (SNP = 74)	0.93 (0.90, 0.95) (<0.001)	737.4 (<0.001)	0.93 (0.89, 0.96) (<0.001)	737.0 (<0.001)	0.850	0.93 (0.92, 0.94) (<0.001)	0.92 (0.91, 0.93) (<0.001)
HDL (SNP = 85)	0.95 (0.91, 0.98) (0.007)	1135.2 (<0.001)	0.90 (0.84, 0.97) (0.006)	1102.1 (<0.001)	0.003	0.92 (0.90, 0.94) (<0.001)	0.92 (0.89, 0.95) (<0.001)

*OR means odds ratio caused by per SD change. MR, Mendelian randomization; LDL, Low-density lipoprotein; HDL, High density lipoprotein; TG, Triacylglycerol*.

**Figure 4:**
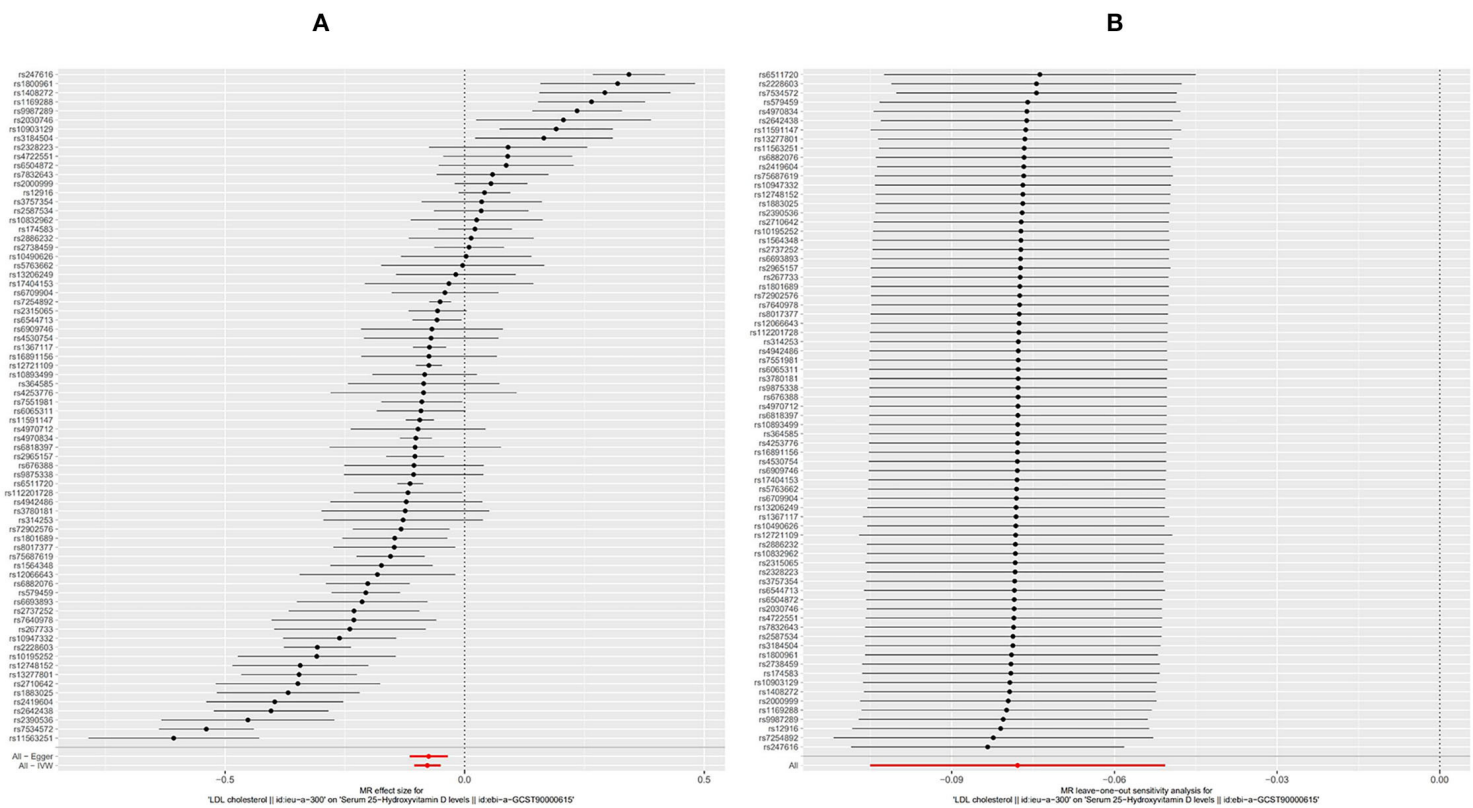
Forest plot of included SNPs in the mendelian randomization analysis for casual effect of low-density lipoprotein (LDL) on vitamin D deficiency. **(A)** Meta-analysis of SNPs' effects on vitamin D deficiency based on inverse variance weighted (IVW) method with random effect model. **(B)** Sensitivity analysis by omitting every SNP. LDL, Low-density lipoprotein.

As for LDL, according to the SVMR analysis result based on the IVM method, elevated serum LDL concentration could cause serum [1,25(OH)2D3] level to also decrease (Figure 4A, OR based on IVW: 0.93, 95%CI:0.90–0.95, *P* < 0.001), sensitivity analysis (IVW method) found that this result was stable (Figure 4B). The Q test also detected significant heterogeneity (Table 2, Q_IVW_ = 737.4, *P* < 0.001). MR-egger Intercept test found that there was no significant pleiotropy in the SVMR between serum LDL and serum [1,25(OH)2D3] (*P*_intercept_ = 0.850). We still performed the analysis based on all four MR methods entirely and found that the relationship between LDL and vitamin D was very stable. Increased LDL serum content would lead to a decrease in serum [1,25(OH)2D3] level (Table 1, OR_MR−Egger_= 0.93, *P* < 0.001 for MR-Egger, OR _WeightedMedian_ = 0.93, *P* < 0.001 for Weighted median, OR_Weightedmode_ = 0.92, and *P* < 0.001 for Weighted mode), a scatter graph was also offered to all four methods (Figure 3B).

**Table 2:** Associations based on multivariable MR (IVW) between genetically predicted circulating lipids and vitamin D levels.

**Lipids**	**OR (95% CI)**	***P*** **-values**
TG (SNP = 435)	0.84 (0.81, 0.87)	<0.001
LDL (SNP = 233)	0.91 (0.89, 0.93)	<0.001
HDL (SNP = 547)	0.90 (0.87, 0.92)	<0.001

*MR, Median Radomization; TG, Triacylglycerol; LDL, Low Density Lipoprotein; HDL, High Density Lipoprotein*.

**Figure 5:**
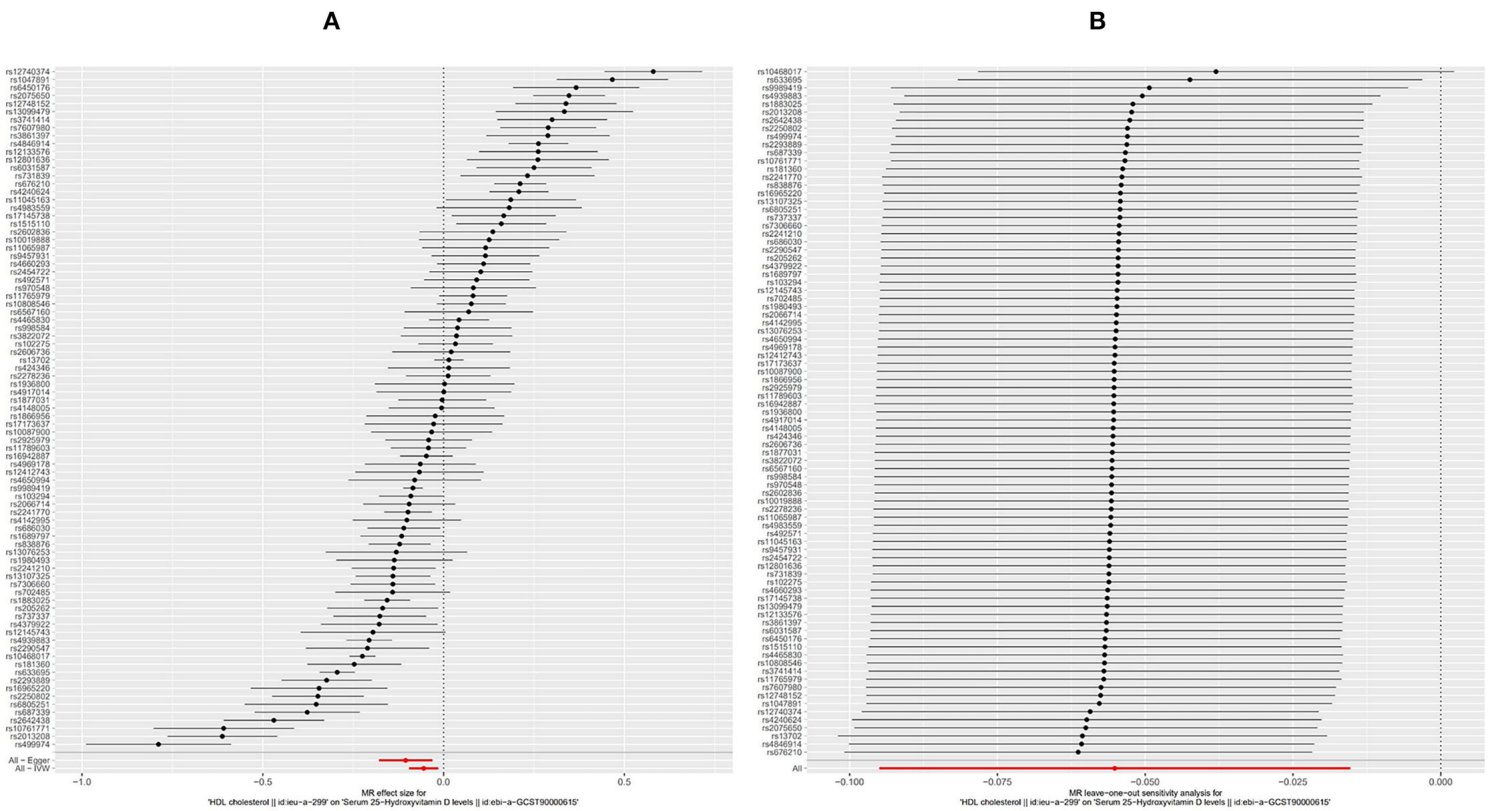
Forest plot of included SNPs in the mendelian randomization analysis for casual effect of HDL on vitamin D deficiency. **(A)** Meta-analysis of SNPs' effects on vitamin D deficiency based on inverse variance weighted (IVW) method with random effect model. **(B)** Sensitivity analysis by omitting every SNP. LDL, High-density lipoprotein.

The HDL showed similar results as TG. First, like the other two substances, an increase in the serum level of LDL will also cause a decrease in the serum level of [1,25(OH)2D3] (Figure 5A, OR based on IVW: 0.95, 95%CI: 0.91–0.98, *P* = 0.007), similar stable sensitivity analysis result (Figure 5B), and significant heterogeneity (Table 2, Q_IVW_ = 1135.2, *P* < 0.001). MR-egger Intercept test found significant pleiotropy in the SVMR between serum TG and serum vitamin D (*P*_intercept_ = 0.003). MR-egger method was applied and found that after the pluripotency adjustment, the causal relationship between elevated TG level and decreased [1,25(OH)2D3] level was still significant (Table 1. MR-egger part, OR: 0.90, 95%CI: 0.84–0.97, *P* = 0.006). The further weighted median MR method (OR: 0.92, 95%CI: 0.89–0.94, *P* < 0.001) and weighted mode MR method (OR: 0.92 95%CI: 0.89–0.95, *P* < 0.001) were also conducted, and all the results were stable (Table 1). A scatter graph was also offered for all four methods (Figure 3C).

Since the effects of the three substances on serum [1,25(OH)2D3] may interfere with each other, further multi-variable MR (MVMR) analysis is necessary. In the MVMR based on GLGC datasets, consistent with the results of SVMR, LDL (*P* < 0.001), HDL (*P* < 0.001), and TG (*P* < 0.001), were all significantly associated with vitamin D deficiency (Table 2).

## Discussion

With the rapid increase in GWAS-related studies in recent years, some GWAS data sets related to vitamin D deficiency have been published. A recent GWAS study found that vitamin D deficiency is significantly related to some SNPs related to lipid metabolism. This has aroused our concern (20, 29). In previous studies, the academic community paid more attention to the impact of vitamin D deficiency on dyslipidemia and less conversely discussed the impact of dyslipidemia on vitamin D (30). The relationship between hyperlipidemia and atherosclerosis has been extensively studied and proven, and the relationship between vitamin D deficiency and atherosclerosis has also been shown to be closely related, and the same situation can also be verified in fracture disease (hyperlipidemia and vitamin D deficiency could both increase the risk of fractures) (31–33). These phenomena pointed out the potential connection between hyperlipidemia and vitamin D deficiency.

According to previous research reports, the main cause of vitamin D deficiency is lack of sun exposure and malnutrition (34, 35). Other risk factors for vitamin D deficiency include inappropriate dietary intake, Zn deficiency, age, increased blood calcium concentration caused by various reasons, etc. (36, 37). An early Mendelian randomization study published in 2013 found that a higher BMI leads to a lower serum [1,25(OH)2D3] concentration, while a lower serum [1,25(OH)2D3] is unlikely to increase BMI. Therefore, the promotion of weight loss programs for obese people may significantly reduce the incidence of [1,25(OH)2D3] deficiency (12). These findings suggest that in patients who are obese or have hyperlipidemia, there may be a mechanism that can lead to vitamin D deficiency.

We found that causal relationship between elevated serum TG, LDL, HDL levels, and decreased serum [1,25(OH)2D3] levels. Significant heterogeneity was found in all three MR analyses, which may affect the stability of the results. In the analysis of TG and HDL, significant pleiotropy was found by the Intercept test. Although the MR-egger method was used to adjust the pleiotropy, the final results still need to be treated with caution. Although there was still a lack of comprehensive molecular biology research, there are still some studies exploring the potential mechanism of vitamin D deficiency caused by hyperlipidemia. A meta-analysis of genetic polymorphisms indicated that CYP2R1 {an important metabolic enzyme in the synthesis of [1,25(OH)2D3] in the liver} mutations in humans are significantly related to vitamin D deficiency (38). Another study conducted on rats found that diet-induced elevation of circulating cholesterol, and glucose could reduce serum [1,25(OH)2D3] levels by suppressing hepatic Cyp2r1 expression (39). As for the specific molecular mechanism research on how hyperlipidemia affects Cyp2r1 expression, it was still lacking.

There were still many limitations in this analysis. First, all the GWAS data were based on the European population (UKB, GLGC cohort). This may lead to biases based on race, environment, and dietary habits. Second, although there is a significant causal relationship between lipids and the occurrence of vitamin D deficiency based on the existing MR analysis results, due to methodological limitations, we cannot rule out the possibility that there are still critically mediating factors between elevated lipids and the occurrence of vitamin D deficiency, such as insulin resistance and many other mechanisms (40).

## Conclusion

We identified that the elevated serum triacylglycerol, LDL, and HDL levels all had a causal relationship with vitamin D deficiency. Taking into account the significant pleiotropy demonstrated in this study, the conclusions of this study should be treated with caution.

## Data Availability Statement

The original contributions presented in the study are included in the article/supplementary material, further inquiries can be directed to the corresponding author/s.

## Ethics Statement

The authors stated that no human studies are presented in the manuscript.

## Author Contributions

ZL, YJ, and JL: conceptualization, methodology, formal analysis, and writing—review and editing. ZL and YJ: writing—original draft preparation. All authors revised it critically for important intellectual content and approved the final version to be published.

## Funding

Grant-in-aid financed this work for scientific research from the Liaoning Province Natural Science Foundation (No. 2020-BS-032).

## Conflict of Interest

The authors declare that the research was conducted in the absence of any commercial or financial relationships that could be construed as a potential conflict of interest.

## Publisher's Note

All claims expressed in this article are solely those of the authors and do not necessarily represent those of their affiliated organizations, or those of the publisher, the editors and the reviewers. Any product that may be evaluated in this article, or claim that may be made by its manufacturer, is not guaranteed or endorsed by the publisher.
